# A Pulmonary Rehabilitation Decisional Score to Define Priority Access for COPD Patients

**DOI:** 10.1155/2017/5710676

**Published:** 2017-01-23

**Authors:** Michele Vitacca, Laura Comini, Marilena Barbisoni, Gloria Francolini, Mara Paneroni, Jean Pierre Ramponi

**Affiliations:** ^1^Respiratory Rehabilitation Division, Istituti Clinici Scientifici Maugeri, IRCCS Lumezzane, Lumezzane, Brescia, Italy; ^2^Health Directorate, Istituti Clinici Scientifici Maugeri, IRCCS Lumezzane, Lumezzane, Brescia, Italy

## Abstract

This retrospective study aimed to evaluate, through an ad hoc 17-item tool, the Pulmonary Rehabilitation Decisional Score (PRDS), the priority access to PR prescription by respiratory specialists. The PRDS, scoring functional, clinical, disability, frailty, and participation parameters from 0 = low priority to 34 = very high priority for PR access, was retrospectively calculated on 124 specialist reports sent to the GP of subjects (aged 71 ± 11 years, FEV_1_%  51 ± 17) consecutively admitted to our respiratory outpatient clinic. From the specialist's report the final subject's allocation could be low priority (LP) (>60 days), high priority (HP) (30–60 days), or very high priority (VHP) (<30 days) to rehabilitation. The PRDS calculation showed scores significantly higher in VHP versus LP (*p* < 0.001) and significantly different between HP and VHP (*p* < 0.001). Comparing the specialist's allocation decision and priority choice based on PRDS cut-offs, PR prescription was significantly more appropriate in VHP than in HP (*p* = 0.016). Specialists underprescribed PR in 49% of LP cases and overprescribed it in 46% and 30% of the HP and VHP prescriptions, respectively. A multicomprehensive score is feasible being useful for staging the clinical priorities for PR prescription and facilitating sustainability of the health system.

## 1. Introduction

Pulmonary rehabilitation (PR) is now an accepted therapy for subjects with respiratory diseases. Its effectiveness is supported by numerous randomized controlled trials. Over the past 30–40 years, PR has evolved from a medical “art” to evidence-based therapy. Several reviews have summarized the evidence for PR [1–4]. From these, the ideal candidate for rehabilitation that emerges is a symptomatic subject with impaired functional status, low participation in activities of daily living (ADLs), and low health-related quality of life (HRQoL), who is a high consumer of healthcare resources and suffering from the systemic (i.e., nonrespiratory) consequences of chronic obstructive pulmonary disease (COPD) [1–5]. Beneficial effects of PR have been demonstrated both in moderate-severe [6–8] and in very mild COPD [9, 10], although a weak recommendation for a routinely PR in the latter has been proposed [11]. Previous research showed also that patients with worse function and health status were the best responders to rehabilitation [6–8]. The positive results of PR are independent of other factors such as age, gender, and smoking status, which are not determinants in predicting the rehabilitation outcome [5].

Otherwise, the current guidelines on PR [1–4] are mainly based on the experience of the programs provided to outpatients and do not offer any multifaceted tool to determine the clear indication for priority of PR prescription. Furthermore, in the current period of economic contraction, health payer needs instruments to define a “priority setting” and “budget impact” to cover costs of PR in order to ensure a better distribution of health resources because we need solid solid instruments and outcomes to decide how to optimally invest money in this field [12, 13].

Clinicians really need to be able to define the priority of PR prescription based on objective and measurable parameters of disease, such as respiratory function, disability, nonparticipation in ADLs, and the complexity of their condition. The development of a dedicated tool could give specialists, general practitioners (GPs), and healthcare providers a common language for both clinical and administrative purposes, enabling them to prioritize PR access.

The aim of this retrospective study was to evaluate (by means of a new tool: Pulmonary Rehabilitation Decisional Score [PRDS], created ad hoc), the appropriateness of priority of PR prescription proposed by specialists in COPD subjects referred to the respiratory outpatients clinic.

## 2. Methods

This study consisted in the development of an assessment decisional tool (PRDS) filled in “a posteriori” by the specialist after the outpatients visit to verify the appropriateness of his decision on priority to PR access. The study was retrospective, approved by our local Scientific Board (CTS 24/11/2015) and by Fondazione S. Maugeri IRCCS Ethics Committee (EC# 2017).

### 2.1. Development of the Pulmonary Respiratory Decisional Score (PRDS)

To reach face validity based on expert opinion involving a structured process of consensus, we enrolled key stakeholders (doctors, nurses, and physiotherapists) from among health staff employed in the rehabilitation field of our Institute. They were invited to participate because their skills and views were considered important. Their participation involved three focus group meetings over a 1-month period. Consent was assumed by the agreement to participate.

**Step 1 .**

We performed a systematic review of the PR literature for COPD identifying patients with specific characteristics and who respond well to PR and prepared a preliminary draft (PRDS) of a priority PR prescription based on a scoring of different items and “cut-offs.”

We created two separate focus groups (Groups 1 and 2), each composed of 3 pulmonologists, 3 physiotherapists, and 2 nurses. The focus group participants were randomly allocated to Group 1 or Group 2. During the first meeting with separated groups, using a Delphi-like procedure, experts were asked to rate the importance of carefully preselected items in the PRDS score on a 5-point Likert scale (0 = absolutely not important; 1 = not important; 2 = minimally important; 3 = important; 4 = very important). Focus group participants were also asked to rank the importance of each item on a scale according to 3 different severity weights for PR priority indication (from 0 = minimum to 2 = maximum priority indication for PR). In general the focus group graded items on the literature's evidence that more severe and disabled patients are more likely to respond to PR. Consensus was considered when more than 75% of the respondents (a) rated each item as mandatory to be inserted in the score when considered “important” or “very important” and (b) agreed to each item's grading from 0 = minimum to 2 = maximum priority indication for PR.

**Step 2 .**

Group 1 and Group 2 received a preliminary feedback by each coordinator (MV, MB) on results of step 1 only relatively to their own group. Then, a combined meeting between Groups 1 and 2 was performed. At this combined meeting, the two focus groups together had the task to do the following: (a) share, compare, confirm, or modify the items and scores proposed, adding any items they felt should be included in the core set which were not on the initial list provided; (b) verify that the items were simple, clear, and comprehensible and if necessary, change them. In particular, focus group discussed if had to have a higher priority for PR referral for smokers with respect to no smoking patients, patients with a worse FEV_1_, patients with severely limited ADL, older patients with respect to younger patients. Focus groups discussed the modality to measure depression/anxiety: in this case groups have preferred to avoid cumbersome questionnaire referring solely to the existing description. Discussion was performed on self-reported adherence to medication/LTOT and self-reported physical activity and on the most appropriate test to measure effort tolerance as 6MWD. Agreement between groups' members was to propose simple information found during a normal face-to-face visit or using previous medical history information so that any operator could easily calculate the new score. In detail, focus group agreed that smoking status is not an exclusion criterion for rehabilitation programs grading this condition more severe than condition of no smoking. Focus group agreed that smoking subjects were the ideal population to invest for behavioral changes in lifestyles offering, at least 1 time in their life, an opportunity to cut the vicious circle related to smoking with a structured educational, psychological, and drug program to quit smoking. The decision to include smoking subjects has been reinforced by our recent experience on smoking as negative predictor for exacerbation [14].

Exacerbation was defined as “an event requiring antibiotics and/or oral steroids as prescribed by specialist or patient's GP” [15] according to GOLD guidelines [16].

Focus group decided to use the number of comorbidities rather than more sophisticated comorbidities score to speed the score report; focus group also decided to grade cardiac and neurological comorbidities in terms of “disability to exercise” as more disabling ones when compared to other comorbidities.

Physical activity profile was obtained by a self-reported answer: “During the last month how much time have you spent weekly for vigorous or moderate activities as gardening, cycling, carrying loads, walking, swimming, performing daily home activity without any kind of help?” Focus group decided that the 6MWT value scheduled was that performed at the time of specialist's visit or the earlier available in the 6 months preceding the specialist's visit.

After discussion in this combined meeting, preliminary conclusions between groups as a whole were reached.

**Step 3 .**

A final plenary consensus meeting was held to present the results from the triage, discuss the measures for the core set, and approve the definitive version of the tool. Final consensus was considered when more than 75% of the respondents rated the PRDS triage acceptable.

The score was thought to be calculated during a specialist visit or assigned to a case manager (nurse or PT) before the medical visit. The time to fill all the items is approximately 5 minutes and the final score is automatically calculated by a dedicated sheet in the Excel program.

### 2.2. Measurements

Data were retrieved from specialist reports compiled at the outpatients visit using (when necessary) hospital report of respiratory functional status and exercise tolerance sent to the GP.

The following data were collected: anthropometrics as age, sex, body mass index (BMI), and smoking status; definitive diagnosis of COPD according to spirometry; clinical status in the previous 6 months (exacerbations and hospitalization), wellbeing or presence of pain; presence of comorbidities; arterial pulsed oximetry (oxygen saturation); present/past data on forced expiratory volume at first second [FEV_1_ (% pred.)] and forced vital capacity [FVC (% pred.)]; dyspnea level measured by the Medical Research Council (MRC) dyspnea scale [17] and disease impact measured by the COPD Assessment Tool (CAT) [18]; weekly physical activity; present/past exercise tolerance measured by the 6-minute walking test (6MWT) [19]; ADL participation; therapy and adherence to treatment; anxiety and depression status; frailty condition with level of care need.

These data were used to retrospectively calculate the PRDS score, which was then compared with the PR priority prescription made by the specialist in the report, as follows: according to our actual regional suggestions (i.e., Lombardy region) low priority (LP) for rehabilitation was defined as PR available over 60 days, high priority (HP) for rehabilitation available within 30–60 days, and very high priority (VHP) for rehabilitation available within 30 days. The PRDS score ranged from 0 = minimum value to 34 = maximum value for priority of PR access.

### 2.3. Subjects

All subjects with chronic obstructive pulmonary disease consecutively referred between 1st January 2016 and 1st March 2016 to the respiratory outpatient clinic of the Istituti Clinici Scientifici Maugeri—IRCCS, Lumezzane (BS), Italy—for a second-opinion specialist consultation (first visit or check-up) were eligible for the study. When admitted in our institution, the patients received an inpatient program with an average stay of 25 (SD 3) days with no less than 22 rehabilitative sessions or an outpatient program attending no less than 22 rehabilitative sessions with 2 or 3 weekly access instances within a 2-month period. During the entire program, interactive and autonomy-supportive approach groups and paper-copy quit strategy and individually tailored quit plan with pharmacotherapy guidance were proposed.

Subjects were excluded if any of the following conditions was present: dyspnea at rest with an urgent need of hospitalization in an acute setting, oncological disease, pain uncontrolled by medication, terminal illness, neuromuscular degenerative diseases, severe orthopedic diseases, subject bedridden or confined to a wheelchair, and altered cognitive status.

### 2.4. Statistical Analysis

To define the concept of PR prescription (i.e., priority values for LP, HP, and VH), 3 ranges of PRDS score were arbitrarily proposed: ≤30% of the maximum PRDS score range (0–10) identified low priority for PR access (PR available over 60 days); between 31% and 50% of max. PRDS score (range 11–17) identified high priority for PR access (PR available within 30 and 60 days); and >51% (range 18–34) of the maximum PRDS value defined a very high priority of PR prescription (PR available within 30 days). PR underprescription was defined by any value outside the range proper to the relative priority allocation, indicating the need for a more urgent allocation. PR overprescription was defined by any value outside the range proper to the relative priority allocation, indicating the need for a less urgent allocation.

Normality of the data was tested by the Shapiro-Wilk test. One-way ANOVA was performed to compare differences among settings and if significant, Bonferroni's multiple comparison was applied as post hoc test. Based on the proposed triage ranges, a test of proportion (test Zeta) was performed to compare differences in the percentage of appropriate prescription, underprescription, and overprescription of PR as decided by the specialist at the outpatient visit. A *p* value < 0.05 was considered statistically significant.

## 3. Results

### 3.1. Development of the PRDS

The first focus group proposed the following clinical items for the PRDS: functional severity (FEV_1_  % pred. and FVC % pred.), dyspnea (MRC score), smoking status, clinical history (exacerbations, hospitalizations), physical activity (hours/week), disability during ADLs, wellbeing, depression and anxiety, comorbidities, age, BMI, frailty (level of care need), and therapy adherence. These items were proposed as mandatory items for the final score (Table 1). Three progressive levels of severity (from 0 = minimal to 2 = maximal priority for PR) were proposed to grade each item, giving a score range which would represent conditions of progressively increasing severity and priority for PR access (Table 1).

The second focus group, after reviewing the proposals, added two new items (exercise tolerance and disease impact), modified the questions for depression and anxiety items, and changed the score weighting for FEV_1_  % pred. and age items.

A definitive 17-item version of the PRDS was finally approved (Table 1). The total score ranged from 0 (minimal priority for rehabilitation) to 34 (maximal priority for rehabilitation).

### 3.2. Retrospective Analysis

One hundred twenty-four outpatient reports (41 indicating LP as a final decision, 37 HP, and 46 VHP) were collected from the hospital database. These reports were the most recent ones available from subjects who fulfilled the selection criteria. In 4 out 124 patients the 6MWD was missing. No missing data were recorded for all other items.

Demographic and clinical characteristics of the study group are presented in Table 2. The majority of COPD patients were elderly; many of them were ex-smokers (49%) or current smokers (19%) with severe lung obstruction, moderate disease impact, and moderate exercise tolerance. According to PR priority prescription made by the specialist in the report (Table 2), very high priority patients were more elderly, with a worse respiratory function and effort tolerance and presenting higher disease impact when compared to high and low priority patients.

**Figure 1:**
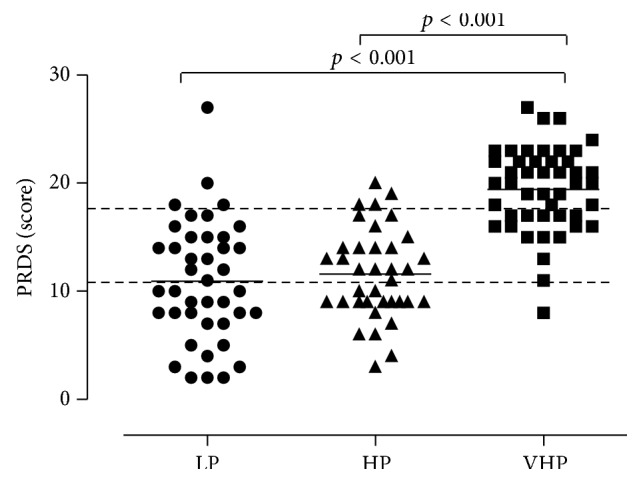
PRDS score, calculated on single report at the time of outpatients visit. Median value indicates the score to be given to the specialist's prescription for low priority (LP, circles, *n* = 41); high priority (HP, triangles, *n* = 37); and very high priority (VHP, squares, *n* = 46). Dotted lines at 11 and 18 delimit the arbitrary cut-offs of PR priority, that is, for LP (0–10), HP (11–17), and VHP (18–34). One-way ANOVA was performed to compare differences among settings and, where significant, Bonferroni's multiple comparison was applied as post hoc test. A *p* value < 0.05 was considered statistically significant.

The PRDS showed a median score of 10 for LP indication, 12 for HP indication, and 20 for VHP indication (Figure 1). As shown in Figure 1, subjects within each PR indication group presented a high variability of PRDS, the coefficient of variation being 51% for LP prescription, 36% for HP, and 20% for VHP. An overlapping of LP and HP prescription was observed. The PRDS score was similar in LP and HP and significantly lower than in VHP prescription (*p* < 0.001 for both) (Figure 1).

**Figure 2:**
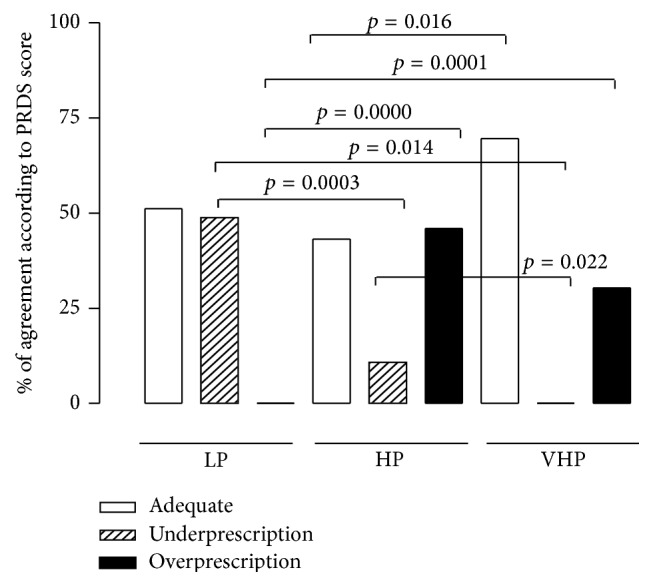
Percentage of adequate prescription (white bars), underprescription (row bars), and overprescription (black bars) of PR rehabilitation in the LP, HP, and VHP groups according to the PRDS cut-off values proposed (LP: 0–10, HP: 11–17, and VHP: 18–34). A test of proportion (test Zeta) was performed to compare differences in the percentage of appropriate prescription, underprescription, and overprescription of PR as decided by the specialist at the outpatient visit. A *p* value < 0.05 was considered statistically significant.

According to the 3 score ranges for priority PR prescription (LP prescription = PRDS 0–10, HP prescription = PRDS 11–17, and VHP prescription = PRDS 18–34) the percentage of subjects who received an adequate priority prescription, underprescription, or overprescription for PR was calculated (Figure 2). Adequate priority prescription was significantly higher (*p* = 0.016) in VHP compared to HP reports; underprescription was significantly higher (*p* < 0.0001) in LP compared to VHP reports; overprescription was significantly higher (*p* = 0.014) in HP compared to LP reports (Figure 2). Of note, specialists at the outpatient visit prescribed low priority rehabilitation (i.e., underestimating the urgency need) in 49% of cases; of this underprescription 75% concerned subjects who would have required PR within 30–60 days and 25% concerned subjects who would have needed PR within 30 days. Otherwise, in 11% of cases specialists prescribed rehabilitation with less urgency than required. Conversely, PR was overprescribed in 46% of HP reports and in 30% of VHP reports.

## 4. Discussion

COPD subjects undergo a gradual decline in respiratory function and their level of disability increases, driving them into a phase of high clinical complexity characterized by repeated exacerbations and hospitalizations. To integrate the multiple dimensions of care necessary for optimal and sustained subject benefits, a “chronic disease care” model [20] has been proposed. PR, with its interdisciplinary patient-centered approach and its emphasis on partnering, communication, and coordination among healthcare professionals, is an excellent platform for the implementation of an integrated chronic disease care model [5].

The issue of appropriateness and suitability of diagnosis, prescription, and care in medicine, particularly in the rehabilitation field, is increasingly being recognized in this period of time when health economic resources are shrinking. For instance, overprescription of PR giving high or very high priority rather than low priority may cause inefficiency, economic waste, and an unjust distribution of health resources.

Future challenges for PR are to find ways to maintain the benefits, promote accessibility to PR, increase the awareness of its value, and install a fair reimbursement scheme [21]. A significant barrier to the effective use of PR in the community is a lack of awareness among many healthcare providers of the nature and benefits of rehabilitation. The difficulty of assessing the real needs of subjects who could benefit most from PR programs makes it impossible to estimate the potential inadequacy between nonexpressed demand and insufficient supply of rehabilitation. As confirmation, the current volume of days devoted to PR represents only 6% of the total rehabilitation days reimbursed by the National Health System in Italy [22]. In real life, evidence of the poor attitude towards PR prescription in Italy was shown in a clinical trial [23] in which only 0.11% of the local population and 2.3% of estimated COPD subjects, in one year, participated in a PR program.

Payment for PR varies widely among healthcare systems worldwide. Reimbursement may represent a gross area of underpayment due to underreporting by hospitals of charges and rehabilitation services required [24] but also a gross overpayment due to an inappropriate choice of candidates for PR and of the setting in which to conduct it.

A multidimensional profiling of response to PR might provide several useful pieces of information, such as the overall priority of intervention, the type of response in each single outcome, and suggestions of how to personalize activities to achieve the goals they perceive as the most important in their daily life [13].

To promote an adequate level of priority in prescription of PR programs, there is a logical need for multidimensional diagnostic, clinical, and functional tools based on multicomprehensive criteria of respiratory function, comorbidities, disability, and frailty. It is reasonable to believe that the efficacy of PR is strictly related to a good subject selection, choosing the patient with the highest potential benefits, and respecting a priority access list. However, in the real world, the choice of the more appropriate subject for PR and the setting in which to conduct PR often depends on funding schemes, the subject's willingness to undergo/continue a program, long waiting lists, socioeconomic reasons, and so on. Hence, the selection of subjects for PR should not be based only on the subject's lung function but rather on a global assessment of the extrapulmonary consequences of COPD, proven to be reversible with rehabilitation [5].

The literature suggests that the ideal candidate for rehabilitation is a symptomatic subject with impaired functional status and low participation in ADLs, high utilization of healthcare resources, and suffering from the systemic consequences of COPD [5].

PR programs have traditionally been developed as outpatient programs. However, in some Italian regions, PR programs are frequently offered in an inpatient setting [22]. Inpatient programs are effective but may be expensive and should thus be reserved perhaps for the more complex subjects. The proposed PRDS score, based on findings from the literature and our own clinical experience, classifies subjects' clinical severity in terms of functional, disability, clinical, psychological, behavioral and frailty, criteria, allowing to better allocate health resources proposing a sort of appropriate priority for PR.

From our data, an adequate PR prescription is clearly more frequent when the indication is for very high priority. Underprescription of PR occurs in 19% of subjects referred to a respiratory specialist (more evident in the group proposed for the low priority list), while overprescription (25% of the whole sample) is more frequent in relation to the high priority admission.

By indicating the appropriate priority for PR the PRDS would (a) facilitate PR prescription to subjects who at present are not given the opportunity and (b) reallocate health costs respecting the priority of PR access.

### 4.1. Study Limitations

The retrospective study design, the ad hoc development of the decisional tree, and the (sometimes) arbitrary choice for the cut points used, without a prospective validation cohort, may be limitations.

No specific additional psychometric testing was conducted as specific reliability, construct validity, and sensitivity and specificity tests.

The outpatient clinic where the study was conducted was located within a rehabilitation hospital and this fact may have undermined the generalizability of results. Nevertheless, we believe that using the PRDS in an in-hospital setting or GP office could reproduce or elicit the present results. The wording “adequate prescription” as used in the paper is based on the scoring and not on quantitative research showing benefits.

We believe the paper raises the issue of an important lack in research, namely, the lack of guidance in the priority prescription of rehabilitation for all chronic respiratory disease patients as well as guidelines about which program/setting suit subject profile. As final limitation we underline that a scoring system, like guidelines, could fall short of addressing the individual patient unique circumstances.

Future studies would establish if actual Pulmonary Rehabilitation Decisional Score could be equally useful for naïve patients or patients with several PR access instances (maintenance rehabilitation).

## 5. Conclusions

In COPD subjects referred to an outpatient respiratory clinic, (i) under- and overprescription of PR occurs and (ii) a new multicomprehensive triage, the Pulmonary Rehabilitation Decisional Score, based on lung function, clinical parameters, disability, frailty, and participation in ADLs, is feasible and may be useful for staging the clinical priority of PR prescription, facilitating the preparation of a waiting list and sustainability of the health system. Future studies will prospectively validate this new tool looking at internal consistency of the items when relating to benefits of PR, PR program completion, comparing triage prescription versus specialist's PR prescription.

## Figures and Tables

**Table 1:** Pulmonary Rehabilitation Decisional Score (PRDS).

ITEMS	Score		
	0	1	2
Age, years	≤59	60–74	≥75
BMI, Kg/m^2^	21–24	25–30	≤20 or ≥31
FEV_1_% pred.	≥65%	36–64%	≤35%
Dyspnea, MRC score (0–4)	0-1	=2	≥3
6MWT, meters	≥350	≤349 and ≥250	≤249
CAT score	≤9	10–15	≥16
Comorbidities	0	1	1 if cardiac/neurological or >1
Activity of daily life	Normal	Limited	Bedridden/wheelchair-restricted
Severe exacerbations in the last year	0	1	>1
Hospitalizations in the last year	0	0, but 1 ER access	>0 or 2 ER access instances
Smoking status	Nonsmoker	Ex-smoker	Current smoker
Physical activity (cyclette, walking, steps)	>4 h/week	2–4 h/week	<2 h/week
Subjective wellbeing	Very well/good	Poor	bad
Depression	No medications	Occasional medications	Under chronic therapy
Anxiety	No medications	Occasional medications	Under chronic therapy
Care need and availability	Not necessary	Useful and available full time	Useful but available on spot or unavailable
Adherence to medications/oxygen	Full	Not constant	Poor/refusal to comply

FEV_1_ = forced expiratory volume at first second; MRC = Medical Research Council; ER = emergency room; W = week; BMI = body mass index; CAT = COPD Assessment Tool; 6MWT = 6-min walking test. Consensus was considered when more than 75% of the respondents (a) rated each item as mandatory to be inserted in the score when considered “important” and/or “very important” and (b) agreed to each item's grading from 0 = minimum to 2 = maximum priority indication for PR. Exacerbation was defined as “an event requiring antibiotics and/or oral steroids as prescribed by specialist or patient's GP.”

**Table 2:** Demographic, anthropometric, and functional characteristics of study patients.

	Whole	LP	HP	VHP	*p*
Patients, *n*	124	41	37	46	
Age, years	71 ± 11	72 ± 12	68 ± 11^&^	74 ± 8	0.031
Males, *n*	73	25	18	30	ns
BMI, Kg/m^2^	24 ± 3	23 ± 2	25 ± 3^$^	24 ± 4	0.0120
Smokers, *n*	24	8	7	9	ns
Ex-smokers, *n*	61	15	19	27	ns
SatO_2_, %	94 ± 2	95 ± 2	94 ± 2°	93 ± 2^*∗*^	<0.0001
FEV_1_, % pred.	51 ± 17	57 ± 15	55 ± 16^?^	43 ± 17^*∗*^	0.0002
FVC, % pred.	78 ± 23	86 ± 19	85 ± 19°	66 ± 23^*∗*^	<0.0001
FEV_1_/FVC%	65 ± 7	65 ± 6	64 ± 9	65 ± 6	ns
CAT, score	14 ± 6	10 ± 4	14 ± 5^∧,&^	17 ± 6^*∗*^	<0.0001
6MWT, meters	321 ± 131	382 ± 144	369 ± 115°	228 ± 62^*∗*^	<0.0001

LP = low priority; HP = high priority; VHP = very high priority. BMI = body mass index; SatO_2_ = pulsed arterial saturation; FEV_1_ = forced expiratory volume at first second; FVC = forced vital capacity; CAT = COPD Assessment Tool; 6MWT = 6-min walking test.  ^$^*p* < 0.05, ^∧^*p* < 0.01, and ^*∗*^*p* < 0.001 versus LP; ^&^*p* < 0.05, ^?^*p* < 0.01, and °*p* < 0.001 versus VHP.

## Abbreviations

ADLs: Activities of daily living
PRDS: Pulmonary Rehabilitation Decisional Score
BMI: Body mass index
COPD: Chronic obstructive pulmonary disease
FEV_1_: Forced expiratory volume
FVC: Forced vital capacity
GPs: General practitioners
HP: High priority
HRQoL: Health-related quality of life
MRC: Medical Research Council
LP: Low priority
VHP: Very high priority
PR: Pulmonary rehabilitation
6MWT: 6-minute walking test.

## Additional Points

*What This Paper Contributes to Our Knowledge*. Pulmonary rehabilitation (PR) is now an accepted therapy for subjects with respiratory diseases. The current guidelines on PR are mainly based on the experience of the programs provided to outpatients and do not offer any multifaceted tool to determine the level of PR priority prescription, for any given subject.* What This Paper Adds*. We have proposed an ad hoc 17-item instrument—the Pulmonary Rehabilitation Decisional Score (PRDS)—to measure the priority appropriateness of PR prescription. In subjects referred to an outpatient respiratory clinic under- and overprescription of PR occurs. The PRDS, based on lung function, clinical parameters, disability, frailty, and participation in ADLs, is feasible and may be useful for staging the clinical priority of PR prescription facilitating sustainability of the health system.
